# Perceived risk and perceptions of COVID-19 vaccine: A survey among general public in Pakistan

**DOI:** 10.1371/journal.pone.0266028

**Published:** 2022-03-24

**Authors:** Bilal Mahmood Beg, Tariq Hussain, Mehmood Ahmad, Sadaf Areej, Arfa Majeed, Muhammad Adil Rasheed, Muhammad Moin Ahmad, Qurat-ul-Ain Shoaib, Sadaf Aroosa

**Affiliations:** 1 Department of Pharmacology and Toxicology, University of Veterinary and Animal Sciences Lahore, Lahore, Pakistan; 2 Department of Basic Sciences, College of Veterinary and Animal Science Jhang, Jhang, Pakistan; 3 Department of Pharmacology, Riphah International University Lahore, Lahore, Pakistan; 4 Department of Community Medicine and Global Health, Institute of Health and Society, University of Oslo, Oslo, Norway; 5 Department of Pharmaceutics, Akhtar Saeed College of Pharmaceutical Science Lahore, Lahore, Pakistan; The University of Mississippi Medical Center, UNITED STATES

## Abstract

**Background:**

The coronavirus disease has become a global pandemic, and it continues to wreak havoc on global health and the economy. The development of vaccines may offer a potential eradication of COVID-19. This study evaluated the general knowledge, attitude, and perception of COVID-19 vaccines in the Pakistani population.

**Methods:**

A self-reporting e-survey and questionnaire-based survey from vaccination centers of different cities of Pakistan among 502 participants were conducted. The questionnaire comprised four sections inquiring demographics, vaccination status, and perception or attitude towards the vaccine. Univariate logistic regression was applied to predict the knowledge, attitude and behavior of participants.

**Results:**

The mean age of participants was 50.8±20.3 years. 53% of the participants have both doses of vaccine administered. Pain on the site of injection (49.8%) was the most common symptom, followed by asthenia (43.0%), muscle pain (29.5%), and swelling (24.5%) on the site of vaccine administration. Females complain of more symptoms than males. More severe symptoms were reported after the first dose of vaccine administration; these symptoms subsided within a week for most participants. Overall, the respondents have a positive attitude towards the vaccine. 47.4% are sure about the vaccine’s efficacy, 48.6% said getting vaccinated was their own decision, and 79.9% also recommended others to get vaccinated.

**Conclusion:**

The study concluded that the Pakistani population has a positive attitude but inadequate knowledge towards COVID-19 vaccines. Immediate awareness and vaccination education programs should be conducted by the authorities to complete the mass vaccination schedule.

## Introduction

The miasmatic coronavirus disease 2019 has adversely affected lives across the globe [1], resulting in 3.29 million deaths among 15 million infected people till May 7, 2021. The mortality ratio increased by 50% in worse affected countries, whereas it was reduced by 5% in countries that observed strict lockdown measures. Among these countries, few reported exact death numbers, whereas in many countries death toll was 1.6 times higher than reported fatalities by the disease [2]. The disease also resulted in the loss of precious time. As per January 2021 report analysis from 81 countries, 20.5 million years of life were lost due to the disease, nine times more than the loss caused by seasonal influenza. Men have lost 45% more life-years than women, with a considerable mortality impact on aged people [3].

In Pakistan, adults aged 19 to 59 were found more susceptible to the disease, with the majority being males due to their social interactions. Another disturbing fact is that health care workers are getting more affected by the infection due to low resources of proper protective equipment. Although COVID-19 patients can easily be recognized by symptoms including fever, cough, and shortness of breath, many people are asymptomatic carriers leading to the faster spread of the virus [4].

Response to a viral infection is either innate or adaptive immune response [5]. When adaptive immune cells (B cells and T cells) encounter the same virus the second time, they clear it before it causes infection. Vaccines use immune memory for protection against the disease produced by either prior infection or an effective vaccine. Two-dose immunity vaccination, which is being used in Pakistan, works by mimicking natural immunity. The first dose triggers initial immune system memory and the second dose petrifies it [6].

The release of the genetic sequence of COVID 19 on January 11, 2020, initiated rapid work on vaccine development to tackle the disease [7]. A total of 115 vaccine applicants were registered in the research and development program, out of which 78 were approved, and 37 were not approved until April 2020. These 73 vaccines went to the preclinical stage for further research. Most advanced were mRNA-1273 from Moderna, Ad5-nCoV from CanSino Biologicals, and INO-4800 from *Inovio*; besides these LV-SMENP-DC and pathogen-specific aAPC from Shenzhen Geno-Immune Medical Institute were also in the run [8].

Pfizer–BioNTech [9] and Moderna [10] developed by the mRNA technique exhibited good efficacy and safety profile. These have also been approved by the US Food and Drug Administration (FDA) and emergency use authorization (EUA) [11]. DNA vaccines are under trial on animals yet [12]. Recombinant protein development is a long-term process. Whole inactivated pathogens and live attenuated vaccines are specific for each infective agent, such as SARS-COV-2.

The whole inactivated virus vaccine prepared by the Beijing Institute of Biological Products is a Sinopharm subsidiary that has gained approval from China’s National Medical Products Agency [13]. This vaccine proved 79.3% effective in the phase 3 trial that gained the focus of the public [14]. The United Arab Emirates became the first country to approve the administration of the Sinopharm vaccine in December 2020 [15]. The Sinovac Corona Vaccine also has gained permission after showing a good safety profile [16]. The CanSino BIO vaccine exhibited 65.7% effectiveness in curing symptomatic patients in its clinical trial resulting in a grant for use in Pakistan by emergency use authorization [17].

Although 1 billion vaccine doses have been administered till May 8 2021, people still hesitate in getting vaccinated due to misconceptions regarding the vaccine. In September 2020, 6.2% of citizens of the UK and 6.4% of citizens of the USA refused to get vaccinated who previously willingly agreed to get the dose. They preferred to get immunity by getting the infection in place of the vaccine due to widespread misconceptions and fake scientific theories regarding vaccines on social networks [18]. Social media is a significant source of information these days. At the beginning of the COVID-19 pandemic majority of the population of Pakistan came to know about the disease either through social media (42.9%) or electronic media (41%) [19]. Numerous false perceptions like infertility in females, hormonal changes and microchip implantation in humans were spread through such mediums that further increased gap between reality and false perception about the vaccine [20]. This study will focus on bridging way toward better understanding of the importance of vaccine and to eradicate rumors by putting forward real views of the public who participated in this study.

Various factors are involved in molding the perceptions of people in Pakistan which may be social, financial or political factors. These factors are involved in influencing perceptions of people regarding the virus and its vaccination [21]. Every other child in Sindh, Pakistan has missed his routine vaccine during the pandemic. Parents are avoiding vaccination for their kids as they believe they may expose their children to virus and may deteriorate their health [22]. Although people are aware of importance of vaccination but still majority of people are confused due to speculations and misconceptions associated with COVID-19 virus and its vaccine [23].

There is an unprecedented need to manufacture and distribute enough safe and effective vaccines to immunize a vast number of individuals to protect the entire global community from the continued threat of morbidity and mortality SARS-CoV-2 [24]. This study aims at understanding people’s perception regarding the disease and vaccine developed to cure it. Results will help to improve understanding and awareness among people regarding importance of vaccine and to eradicate misconceptions associated with the vaccination programs.

## Materials and methods

This questionnaire-based study was carried out in the month of April 2021. A questionnaire was designed and sent out to 50 academicians working in various capacities in higher education institutions. Their responses and suggestions were evaluated, and the questionnaire was modified accordingly. The questionnaire comprised of close-ended questions, and in some of the questions, an option was provided to the respondents to add any other choice if they wanted to provide extra information. The first section of the form comprised of demographics of the respondents. The second section of the form contained the questions related to the vaccine and whether the participants had had a dose of vaccine administered. The next section was added to gather the information regarding the traveling habits of respondents. The fourth section inquired about the information of signs, symptoms or adverse effects following the COVID-19 vaccine administration. The final section of the form was aimed to assess the general perception of the participants towards the COVID-19 vaccine. The google forms were sent out through emails. Also, the data was collected from the various vaccine administration centers working all across Pakistan to ensure the randomness of the data. After permission from local supervisors of the respective vaccination center, our representatives collected the data from participants at the reception of the vaccination center. The participation of the respondents were completely voluntary and they were explained the purpose of the study before the questionnaire was filled out. The participants were informed of possible publication and sharing of the data in future with concealed identity and they were given a choice that they may leave the form any time without submitting the form. The authors made sure that the data shared was fully anonymous. No minors were included in the study as the study was only limited to the responses of individuals above 18 years. Furthermore, at the time the study was conducted, the vaccination of individuals under 18 years of age had not started in Pakistan. An ethical approval for the study was granted by the Institutional Ethics Committee (IEC) of Riphah International University (Letter No. RCVETS-780; Dated: 28-06-2021).

Forms with incomplete data sets or vague responses were removed from the study. 1500 forms were distributed among general public by convenience sampling method with an aimed confidence interval of around 97%. A total of 763 forms were received, out of them, 655 forms were received online whereas 108 forms were collected from the vaccination centers. The sample size was estimated using an online tool (http://www.raosoft.com/samplesize.html). The final sample size has the margin of error of 3.48% (CI: 96.52%). The first few questions of the questionnaire asked the respondents regarding their demographics while the next question asks the participants whether they have received the vaccine. If an individual selected a “No” response, then the form will submit and no further questions were asked. In contrast, if a respondent selected a “Yes” response then the respondents may continue with the next of the questions. Since the participation in the study was completely voluntary that is why a participant was given a choice to leave the form if he or she did not want to fill out the form completely. The raw data was compiled in.csv format and was evaluated for data completeness.

The data was exported to the Statistical Package for the Social Sciences version 21(SPSSv21) for statistical analysis. The results were presented as mean, interquartile ranges (IQR), and percentages. A Pearson chi-square test was applied to evaluate the relationship between the different variables. Furthermore, univariate regression analysis was carried out to ascertain the likelihood of relationships between independent and dependent variables of the study.

## Results and discussion

A total of 763 responses were received out of 1500, making a response rate of 50.87%. Out of 763, a total of 234 participants replied that they have not been vaccinated and hence were excluded from the study. Similarly, 27 respondents were excluded on the failure of data completeness. Finally, only 502 patients were left for further data analysis. 277 (55.2%) male respondents took part in the study while the rest of the respondents were female (44.8%). The mean age of participants were 50.8±20.3 years, with age range of 18 years to 89 years. The demographic section of the questionnaire was stratified as per the eligibility criteria of the vaccine preference designated by the CDC [25]. Phase 1a comprises the professions that are directly in contact with the COVID-19 patients. This includes medical and paramedical staff working in Government or private settings. Phase 1b professions include pertinent manufacturing industries as well as the first responders of law and order professions. Whereas Phase 1c includes other professions and individuals with low or minimum public contact. In our study, we observed that the highest number (42.2%) of vaccine recipients were from Phase 1a followed by Phase 1c (39.2%) and at last Phase 1b (18.5%). A considerable number of the participants (59.8%) belonged to Punjab, the most densely populated province of Pakistan. Whereas the percentage of participants from Azad Jammu & Kashmir (AJK), Islamabad, Sindh, Khyber Pakhtunkhwa (KPK), Baluchistan, and Gilgit-Baltistan (GB) was 10.8%, 10.6%, 8.8%, 8.0%, 1.6%, and 0.6%, respectively. Most of the participants of the study were educated (83.5%). Most of the respondents are university graduates (64.9%). Out of them, 34.7% had an undergraduate level degree, whereas 17.7% and 12.5% participants had a master’s and doctorate, respectively. Only 25.1% participants had early school education where as 5.8% had education till intermediate. In a study conducted in Sindh, Pakistan it was ascertained that misconceptions about side effects of the vaccine were more prevalent among uneducated people compared to educated individuals. 59.9% participants with majority illiterate population believed that getting vaccinated will to development of severe side effects. Vaccine acceptance has been a huge challenge in Pakistan even for polio, measles and mumps due to poor understanding and misconceptions related to vaccines in Pakistan [23].

The highest number of vaccine recipients were employed (45.2%), followed by the group of retired (20.1%). This trend was followed by housewives (14.9%), unemployed participants (7.8%), students (6.2%), and businesspersons (5.8%) (Table 1).

**Table 1:** Demographics of the participants.

Characteristic	Total	Phase 1a	Phase 1b	Phase 1c
	*N = 502*	*n = 212 (42*.*2)*	*n = 93 (18*.*5)*	*n = 197 (39*.*2)*
**Gender**				
***Male***	277 (55.2)	99 (19.7)	75 (14.9)	103 (20.5)
***Female***	225 (44.8)	113 (22.5)	18 (3.6)	94 (18.7)
**Province**				
***Punjab***	300 (59.8)	115 (22.9)	50 (10.0)	135 (26.9)
***Sindh***	44 (8.8)	16 (3.2)	12 (2.4)	16 (3.2)
***Gilgit-Baltistan (GB)***	3 (0.6)	1 (0.5)	2 (0.4)	0 (0.0)
***Islamabad***	53 (10.6)	33 (6.6)	10 (2.0)	10 (2.0)
***Khyber Pakhtunkhwa (KPK)***	40 (8.0)	9 (1.8)	8 (1.6)	23 (4.6)
***Azad Jammu & Kashmir (AJK)***	54 (10.8)	34 (6.8)	9 (1.8)	11 (2.2)
***Baluchistan***	8 (1.6)	4 (0.8)	2 (0.4)	2 (0.4)
**Education**				
***Under matriculation***	83 (16.5)	0 (0.0)	4 (0.8)	79 (15.7)
***Matriculation***	43 (8.6)	1 (0.2)	2 (0.4)	40 (8.0)
***Intermediate***	29 (5.8)	5 (1.0)	7 (1.4)	17 (3.4)
***Diploma***	21 (4.2)	6 (1.2)	11 (2.2)	4 (0.8)
***Bachelor’s Degree***	174 (34.7)	88 (17.5)	31 (6.2)	55 (11.0)
***Master’s Degree***	89 (17.7)	66 (13.1)	22 (4.4)	1 (0.2)
***Doctorate***	63 (12.5)	46 (9.2)	16 (3.2)	1 (0.2)
**Work Status**				
***Employed***	227 (45.2)	172 (34.3)	44 (8.8)	11 (2.2)
***Own a business***	29 (5.8)	8 (1.6)	3 (0.6)	18 (3.6)
***Student***	31 (6.2)	15 (3.0)	13 (2.6)	3 (0.6)
***Housewife***	75 (14.9)	0 (0.0)	1 (0.2)	74 (14.7)
***Unemployed***	39 (7.8)	9 (1.8)	3 (0.6)	27 (5.4)
***Retired***	101 (20.1)	8 (1.6)	29 (5.8)	64 (12.7)

Out of 763 participants, 30.6% participants didn’t get any vaccination dose and were excluded from the study. A study suggests 70% or less population among Africa, Middle East and some European nations hesitate in getting vaccinated [26]. Another Italian university study suggested 13.9% students were reluctant at getting vaccinated instead they preferred learning and adopting healthy habits [27]. In an Egyptian medical college 90.5% students understood importance of vaccine but had serious concerns about effectiveness and side effects of the vaccine [28]. These researches clearly suggest that people with poor understanding about the disease and vaccine prefer to avoid the vaccination dose whereas educated population understands its importance.

In the second section of the questionnaire, the participants were asked about the vaccine doses that they have received. 46.8% of the participants had the first dose of the vaccine only, whereas the 53.2% of respondents had both doses of vaccine administered. When stratified according to gender, it was observed that 24.5% of males and 22.3% of females had the first dose of vaccine. Similarly, 30.7% of males and 22.5% of females had both doses of vaccine administered. However, there was no significant difference in vaccine administration for each gender. 16.5% of the vaccine recipient were previously infected with COVID-19. On the contrary, nearly 66.5% of the individuals did not contract COVID-19 in the past. Furthermore, 16.9% of the participants were not sure whether they had been previously infected or not.

**Fig 1:**
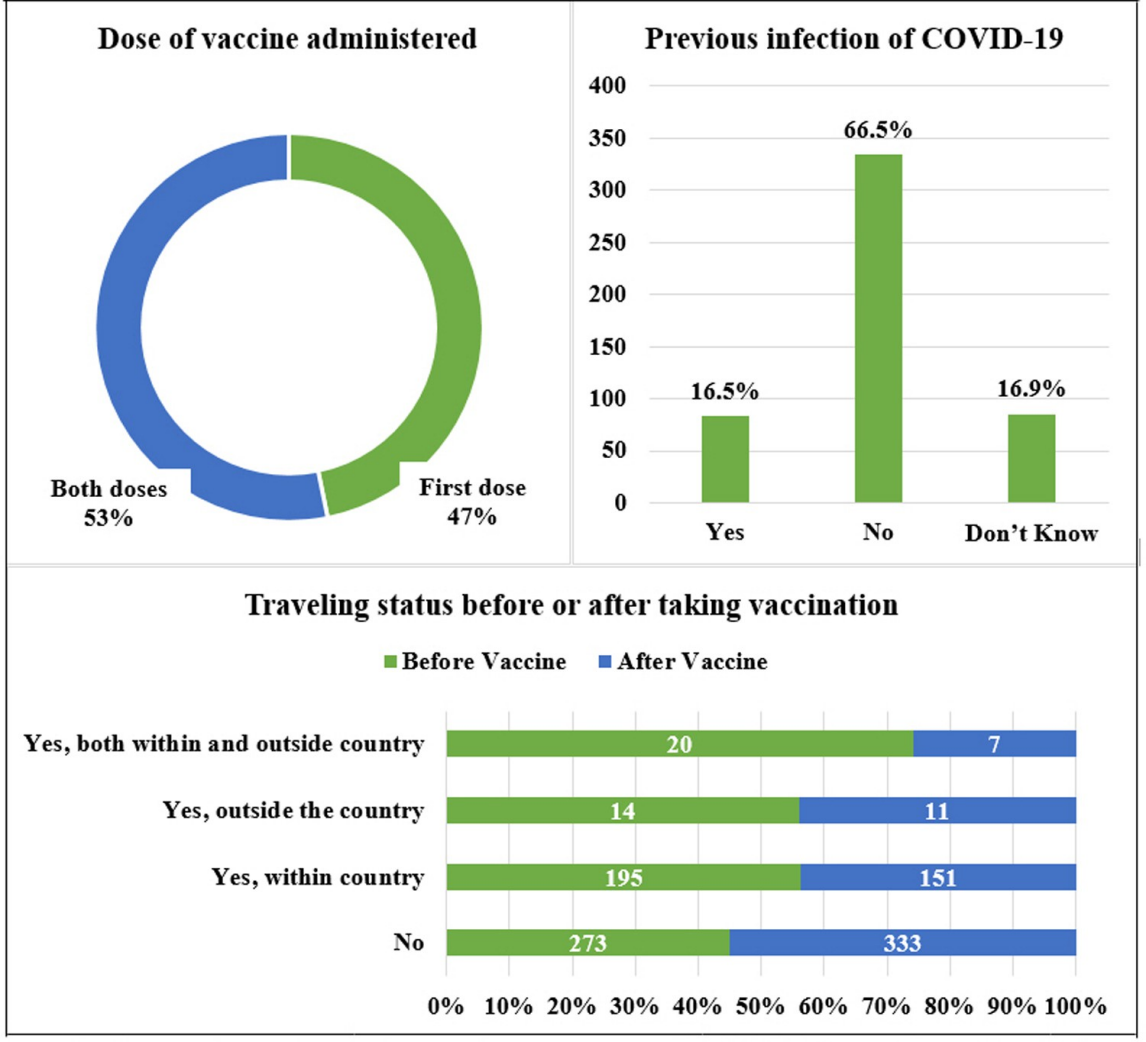
Current vaccine status, past occurrence of COVID-19, and traveling history of respondents.

We also asked the respondents whether they had traveled before taking the vaccination. 54.4% of participants responded that they had not traveled before taking vaccination, whereas 38.8% of the participants traveled within the country, 2.8% of the participants traveled outside the country only, and the rest 4.0%, had traveled both within the country and outside the country (Fig 1). Among the respondents traveling, we further inquired how often they have traveled. We found out that most of the participants (24.9%) had traveled more than twice, 13.1% traveled only once, and 7.6% had commuted twice.

The third section of the survey was designed to get insights into the co-morbidities, medical conditions, and the effects of the vaccines that the participants experienced. In the first question of this section, the respondents were asked if they had any medical condition from the given options. The participants may choose single or multiple options for this question. 68.3% (males = 37.3% and females = 31.1%) of the total population had no co-morbid condition. In contrast, 31.7% (17.7% males and 13.9% females) of the total participants responded that they have an illness that may aggravate the COVID-19. Hypertension (16.3%) was the most prevalent condition among the participants, followed by diabetes (13.5%), obesity (6.8%), asthma (2.6%), arthritis or joint pain (2.4%). Other participants reported dust or pollen allergies (1.2%) and dementia (1.6%). More females (5.2%) had obesity as compared to the males (1.2%) (P<0.001). The next question inquired regarding the smoking status of the participants. Only 3.2% of the male participants were found to be a smoker (P<0.001).

A cross sectional study in Czech Republic reported pain at site of injection, fatigue, headache, muscle pain and chills as most prevalent side effects of the vaccine which lasted for 1–3 days. Besides this people with both doses of vaccine reported more side effects then people who got just a single dose [29]. The candidates were further asked whether they had experienced any signs or symptoms after the vaccine administration. 55.8% of the total participants reported that they had experienced symptoms after the vaccine administration, whereas 44.2% did not experience any of the symptoms. When stratified according to gender, it was observed that females (28.3%) had experienced significantly more signs and symptoms as compared to males (27.5%) (P<0.05). The symptoms were categorized into three different classes: 1) the signs or symptoms that appeared on the site of injection i.e., pain, swelling, redness; 2) the signs or symptoms experienced in the whole of the body; 3) any rare signs or symptoms. 51.0% of the total participants reported that they had experienced unpleasant signs on the site of vaccine administration. The pain was the most commonly reported event among 49.8% of the participants, swelling on the injection site was the second (24.5%) most frequently reported sign, and 13.3% of the respondents experienced redness. Overall, females experienced more signs and symptoms on the injection site than males (P<0.001). Similarly, pain and swelling were most reported by females than males (P<0.05). The next part of the question analyzed for the signs or symptoms experienced in the whole body and 55.4% reported that they have experienced any of the problems. Once again, in general, females (28.1%) reported significantly more signs and symptoms in the body as compared to males (27.3%) (P<0.05). Asthenia or tiredness or body weakness was the most reported symptom (43.0%) by males (21.5%) and females (21.5%) equally; however, females reported significantly more symptoms of asthenia (P<0.05). Muscle pain (29.5%) was the second most common, followed by vertigo (13.9%), nausea or vomiting (9.8%), headache (8.4%), fever (6.6%), and sore throat (6.0%). Fever and nausea are significantly more prevalent in males as compared to females (P<0.05), whereas asthenia and stomach upset are more prevalent among females (P<0.05). Only 10% of the total participants responded that they had experienced rare symptoms. Depression (5.2%), anxiety (5.2%), and mood swings (5.2%) were the most commonly reported rare symptoms. No significant difference was observed between male and female responses regarding rare symptoms.

The next question was the continuity of the previous questions, and the respondents were inquired if their illness is affected by the COVID-19 vaccination. 65.5% of the total participants were completely healthy and had no prior disease. 27.9% reported that the vaccine did not aggravate their illness whereas 6.6% of participants reported that their illness increased after the vaccine administration. When stratified according to gender, more males reported that their illness aggravated after a vaccine than the females (P<0.05). On the contrary, females reported that their disease was not affected after the administration of the vaccine (P<0.05). In addition to this question, another question asked for the percentage of the illness aggravated after the vaccine. 5.8% of individuals responded that their illness increased less than half, whereas 0.8% responded that they had experienced more than 50% increase in the disease. Females reported more than males that their illness increased more than half (P<0.05).

The participants were asked which dose of vaccine resulted in the severe symptoms. Most of the respondents (47.6%) pointed out that none of the vaccine doses resulted in symptoms. On the other hand, 37.8% of the participants reported that they had experienced most of the symptoms after 1^st^ dose of the vaccine. Only 13.7% of the total reported severe symptoms after 2^nd^ dose, and 0.8% replied that both the doses of vaccine resulted in severe symptoms. 20.5% of the participants recovered entirely from the symptoms within a week, 7.8% recovered within two weeks, 2.0% recovered within a month, and 2.2% individuals recovered from the symptoms after a month (Table 2).

**Table 2:** Presence of comorbidities, signs, and symptoms associated with vaccine administration.

	Total	Male	Female	P-value
**Did you have any medical condition that may aggravate COVID-19 illness**	**159 (31.7)**	**89 (17.7)**	**70 (13.9)**	**P>0.05**
***Diabetes***	*68 (13*.*5)*	*41 (8*.*2)*	*27 (5*.*4)*	*P>0*.*05*
***Hypertension***	*82 (16*.*3)*	*47 (9*.*4)*	*35 (7*.*0)*	*P>0*.*05*
***Arthritis/Joint Pain***	*12 (2*.*4)*	*8 (1*.*6)*	*4 (0*.*8)*	*P>0*.*05*
***Obesity***	*34 (6*.*8)*	*8 (1*.*6)*	*26 (5*.*2)*	*P<0*.*001*
***Asthma***	*13 (2*.*6)*	*6 (1*.*2)*	*7 (1*.*4)*	*P>0*.*05*
***Allergies***	*6 (1*.*2)*	*5 (1*.*0)*	*1 (0*.*2)*	*P>0*.*05*
***Dementia***	*8 (1*.*6)*	*5 (1*.*0)*	*3 (0*.*6)*	*P>0*.*05*
***Other conditions***	*14 (2*.*8)*	*7 (1*.*4)*	*7 (1*.*4)*	*P>0*.*05*
***None***	*343 (68*.*3)*	*187 (37*.*3)*	*156 (31*.*1)*	*P>0*.*05*
**Smoking Status**				
***Non Smoker***	*486 (96*.*8)*	*261 (52*.*0)*	*225 (44*.*8)*	*P>0*.*05*
***Smoker***	*16 (3*.*2)*	*16 (3*.*2)*	*0 (0*.*0)*	*P<0*.*001*
**Did you experience any symptoms after vaccination administration?**				
***No***	*222 (44*.*2)*	*139 (27*.*7)*	*83 (16*.*5)*	
***Yes***	*280 (55*.*8)*	*138 (27*.*5)*	*142 (28*.*3)*	*P = 0*.*003*^*a*^
**On site of injection**	**256 (51.0)**	**121 (24.1)**	**135 (26.9)**	**P<0.001** ^**a**^
***Pain***	*250 (49*.*8)*	*117 (23*.*3)*	*133 (26*.*5)*	*P<0*.*001*^*a*^
***Swelling***	*123 (24*.*5)*	*58 (11*.*6)*	*65 (12*.*9)*	*P = 0*.*039*^*a*^
***Redness***	*67 (13*.*3)*	*34 (6*.*8)*	*33 (6*.*6)*	*P>0*.*05*
**In Whole Body**	**278 (55.4)**	**137 (27.3)**	**141 (28.1)**	**P = 0.003** ^**a**^
***Sore throat***	*30 (6*.*0)*	*15 (3*.*0)*	*15 (3*.*0)*	*P>0*.*05*
***Headache***	*42 (8*.*4)*	*18 (3*.*6)*	*24 (4*.*8)*	*P>0*.*05*
***Asthenia***	*216 (43*.*0)*	*108 (21*.*5)*	*108 (21*.*5)*	*P = 0*.*043*^*a*^
***Muscle Pain***	*148 (29*.*5)*	*72 (14*.*3)*	*76 (15*.*1)*	*P>0*.*05*
***Chills***	*6 (1*.*2)*	*2 (0*.*4)*	*4 (0*.*8)*	*P>0*.*05*
***Fever***	*33 (6*.*6)*	*26 (5*.*2)*	*7 (1*.*4)*	*P = 0*.*005*^*b*^
***Nausea/Vomiting***	*49 (9*.*8)*	*19 (3*.*8)*	*30 (6*.*0)*	*P = 0*.*015*^*b*^
***Stomach Upset***	*29 (5*.*8)*	*6 (1*.*2)*	*23 (4*.*6)*	*P<0*.*001*^*a*^
***Vertigo***	*70 (13*.*9)*	*39 (7*.*8)*	*31 (6*.*2)*	*P>0*.*05*
***Restlessness***	*3 (0*.*6)*	*0 (0*.*0)*	*3 (0*.*6)*	*P>0*.*05*
***Dry throat***	*5 (1*.*0)*	*2 (0*.*4)*	*3 (0*.*6)*	*P>0*.*05*
**Quite Rare Symptoms**	**50 (10.0)**	**31 (6.2)**	**19 (3.8)**	**P>0.05**
***Depression***	*26 (5*.*2)*	*19 (3*.*8)*	*7 (1*.*4)*	*P>0*.*05*
***Anxiety***	*26 (5*.*2)*	*19 (3*.*8)*	*7 (1*.*4)*	*P>0*.*05*
***Mood swing***	*26 (5*.*2)*	*19 (3*.*8)*	*7 (1*.*4)*	*P>0*.*05*
***Rash***	*8 (1*.*6)*	*2 (0*.*4)*	*6 (1*.*2)*	*P>0*.*05*
***Breathing issues***	*3 (0*.*6)*	*3 (0*.*6)*	*0 (0*.*0)*	*P>0*.*05*
***Hair loss***	*8 (1*.*6)*	*2 (0*.*4)*	*6 (1*.*2)*	*P>0*.*05*
***CVD***	*3 (0*.*6)*	*2 (0*.*4)*	*1 (0*.*2)*	*P>0*.*05*
***Sleep disturbance***	*3 (0*.*6)*	*1 (0*.*2)*	*2 (0*.*4)*	*P>0*.*05*
***Loss of concentration***	*3 (0*.*6)*	*1 (0*.*2)*	*2 (0*.*4)*	*P>0*.*05*
**The illness affected after COVID-19 vaccination**				
***No prior disease/Completely Healthy***	*329 (65*.*5)*	*196 (39*.*0)*	*133 (26*.*5)*	*P = 0*.*009*^*b*^
***Illness not affected at all***	*140 (27*.*9)*	*62 (12*.*4)*	*78 (15*.*5)*	*P = 0*.*009*^*a*^
***Yes*, *illness aggravated after vaccine***	*33 (6*.*6)*	*19 (3*.*8)*	*14 (2*.*8)*	*P = 0*.*009*^*b*^
**How much do you think your illness increased?**				
***Less than 50%***	*29 (5*.*8)*	*16 (3*.*2)*	*13 (2*.*6)*	*P>0*.*05*
***More than 50%***	*4 (0*.*8)*	*3 (0*.*6)*	*1 (0*.*2)*	*P = 0*.*019*^*a*^
**Dose of vaccine which resulted in more severe symptoms**				
***First dose***	*190 (37*.*8)*	*94 (18*.*7)*	*96 (19*.*1)*	*P>0*.*05*
***Second dose***	*69 (13*.*7)*	*39 (7*.*8)*	*30 (6*.*0)*	*P>0*.*05*
***Both doses***	*4 (0*.*8)*	*3 (0*.*6)*	*1 (0*.*2)*	*P>0*.*05*
***None of them***	*239 (47*.*6)*	*141 (28*.*1)*	*98 (19*.*5)*	*P>0*.*05*
**How many days later you recovered completely from symptoms?**				
***Within 1 week***	*103 (20*.*5)*	*53 (10*.*6)*	*50 (10*.*0)*	*P>0*.*05*
***Within 2 weeks***	*39 (7*.*8)*	*18 (3*.*6)*	*21 (4*.*2)*	*P>0*.*05*
***Within 4 weeks***	*10 (2*.*0)*	*4 (0*.*8)*	*6 (1*.*2)*	*P>0*.*05*
***More than a month***	*11 (2*.*2)*	*7 (1*.*4)*	*4 (0*.*8)*	*P>0*.*05*

The last section of the questionnaire looked for the responses of individuals based on their thoughts and post-vaccine practices. Firstly, we asked them that how many family members from their family had been vaccinated. 42.0% of the responses were that only older people among the family are vaccinated only. 24.3% replied that none of their family members had been vaccinated so far. 15.5% responded that all of their family members had been vaccinated. However, some individuals (14.7%) responded that only medical professionals had been vaccinated in their families. Similarly, only 3.4% of the total interviewees replied that only medical professionals and old-age people were vaccinated in their families. Another cross sectional study suggest that government policy of free of cost vaccination for older people resulted in 62% population accepting vaccination regardless of false theories related to the vaccine [30].

When asked regarding post-vaccine travel, we observed that 30.1% of the individuals traveled within the country, 2.2% traveled outside the country, and 1.4% traveled both within and outside the country. However, it was observed that post-vaccine travel has decreased than pre-vaccine times. Furthermore, we asked respondents if the vaccine was effective. 47.4% of the individuals were entirely sure about its efficacy. However, 31.5% replied that they are just following others, 17.9% think the vaccine is ineffective, and 3.2% are unsure. Males were significantly more confident than females regarding the efficacy of vaccination *(P = 0*.*02)*. Getting a vaccination jab is the decision of most of the individuals (48.6%); 30.1% of the participants responded that the decision of getting vaccinated is the decision of their family members. 20.3% of the individuals reported that vaccine was their workplace requirement. 71.1% of the participants claimed that they had complete information on the vaccine. Most of the people (39.2%) think that the COVID-19 epidemic is going to stay here. On the brighter side, 27.1% of the participants think that this pandemic will end by the end of this year. 79.9% of the participants encouraged others to get vaccinated, whereas 19.3% thought that one may only get vaccinated if they need it (Table 3). Acceptance of vaccine is increasing with better understanding of the virus and its vaccine with young healthcare worker having highest acceptance for the vaccine jab [17].

**Table 3:** Perception towards vaccine.

	Total	Males	Females	P-value
**Family members vaccinated?**				
***None***	*122 (24*.*3)*	*70 (13*.*9)*	*52 (10*.*4)*	*P>0*.*05*
***Yes*, *everyone***	*78 (15*.*5)*	*38 (7*.*6)*	*40 (8*.*0)*	*P>0*.*05*
***Yes*, *old people only***	*211 (42*.*0)*	*120 (23*.*9)*	*91 (18*.*1)*	*P>0*.*05*
***Yes*, *medical professionals only***	*74 (14*.*7)*	*41 (8*.*2)*	*33 (6*.*6)*	*P>0*.*05*
***Medical professionals and old age only***	*17 (3*.*4)*	*8 (1*.*6)*	*9 (1*.*8)*	*P>0*.*05*
**Thoughts if the vaccine is effective?**				
***No*, *it may not be effective***	*90 (17*.*9)*	*39 (7*.*8)*	*51 (10*.*2)*	*P = 0*.*02*^*a*^
***Following others***	*158 (31*.*5)*	*82 (16*.*3)*	*76 (15*.*1)*	*P = 0*.*02*^*b*^
***Not sure***	*16 (3*.*2)*	*10 (2*.*0)*	*6 (1*.*2)*	*P = 0*.*02*^*a*^
***Completely sure about its efficacy***	*238 (47*.*4)*	*146 (29*.*1)*	*92 (18*.*3)*	*P = 0*.*02*^*b*^
**Decision of vaccine:**				
***Own decision***	*244 (48*.*6)*	*151 (30*.*1)*	*93 (18*.*5)*	*P = 0*.*043*^*b*^
***Workplace requirement***	*102 (20*.*3)*	*48 (9*.*6)*	*54 (10*.*8)*	*P = 0*.*043*^*a*^
***Family wanted me to get vaccinated***	*151 (30*.*1)*	*75 (14*.*9)*	*76 (15*.*1)*	*P = 0*.*043*^*a*^
***Moving out of country***	*1 (0*.*2)*	*1 (0*.*2)*	*0 (0*.*0)*	*P = 0*.*043*^*b*^
***All of the above***	*4 (0*.*8)*	*2 (0*.*4)*	*2 (0*.*4)*	*P = 0*.*043*^*b*^
**Complete information on the vaccine?**				
***Yes***	*357 (71*.*1)*	*202 (40*.*2)*	*155 (30*.*9)*	*P>0*.*05*
***No***	*145 (28*.*9)*	*75 (14*.*9)*	*70 (13*.*9)*	*P>0*.*05*
**When will the COVID-19 epidemic end?**				
***End of this year***	*136 (27*.*1)*	*79 (15*.*7)*	*57 (11*.*4)*	*P = 0*.*023*^*b*^
***Going nowhere***	*197 (39*.*2)*	*119 (23*.*7)*	*78 (15*.*5)*	*P = 0*.*023*^*b*^
***Don’t know***	*169 (33*.*7)*	*79 (15*.*7)*	*90 (17*.*9)*	*P = 0*.*023*^*b*^
**Would you recommend others to get vaccinated?**				
***Yes***	*401 (79*.*9)*	*226 (45*.*0)*	*175 (34*.*9)*	*P>0*.*05*
***No***	*4 (0*.*8)*	*2 (0*.*4)*	*2 (0*.*4)*	*P>0*.*05*
***Only if needed***	*97 (19*.*3)*	*49 (9*.*8)*	*48 (9*.*6)*	*P>0*.*05*

^a^ Females experienced more symptoms as compared to males

^b^ Males experienced more symptoms as compared to females

In addition to the results given above, we also carried out univariate logistic regression to evaluate the effect of age and other independent factors on the attitude or responses of the participants. Firstly, logistic regression was carried out to ascertain the impact of age on the likelihood of the symptoms after vaccination. Increasing age was found to be positively correlated with more symptoms after vaccination (P<0.001). Similarly, increasing age was associated with an increased likelihood of exhibiting symptoms at the site of injection (P<0.001) and in the whole body (P<0.001). No relationship was found between the previous COVID-19 infection and the occurrence of the symptoms after vaccine administration (P>0.05). Similarly, the smoking status and disease condition of a participant was not positively associated with the presence of side effects of vaccination (P>0.05). Furthermore, the presence of a comorbid disorder was more related to the exhibition of rare symptoms following a vaccine (P<0.001); however, the comorbidity did not result in the occurrence of injection site and whole-body side effects. Moreover, when hypertension, diabetes, asthma, other conditions were evaluated against the overall symptoms, it was observed that none of the conditions alone significantly affected the occurrence of signs or symptoms. Only the first dose of vaccine has resulted in significantly more pain, swelling, or redness at the site of injection (P<0.05) as compared to the second dose of vaccine. The development of vaccines to put the spread of the COVID-19 virus at a halt had been an imperative strategy ever since its first outbreak. More than 100 potential COVID-19 vaccines were being developed as of March 2020, and thirteen have been approved [31]. This study sought to evaluate the Pakistani population’s awareness and acceptance of COVID-19 vaccines once they became commercially available in the country. Understanding the vaccine’s importance in Pakistan is vital because the country has a large population with a high disease burden and relatively high vaccine hesitancy, and low vaccine coverage [32]. Therefore, it is necessary to understand the acceptance rate and behavior of the general population to eradicate any barriers in the way of vaccination.

Our study finds out that the highest number of vaccine recipients were the professionals directly in contact with the COVID-19 patients, followed by individuals with low or minimum public contact. Lastly, professionals with indirect contact with COVID-19 patients received the least number of vaccines. This distribution pattern occurred because, in Pakistan, the healthcare workers were priority then the vaccine was rolled out to the older patients and finally to other professionals, students, and unemployed citizens [33]. A higher acceptance towards vaccine was observed in females who have also been reported in other studies [34, 35]; however, a contrast in literature also exists where the acceptance rate in males is otherwise higher than females [36].

Although 503 people participated in the study but 59.8% population belonged to Punjab who had good academic record. The study also indicates that a higher population of respondents were fairly educated and belonged to Punjab. This could be due to the reason that Punjab is the most densely populated province with a higher literacy rate than other provinces. Moreover, the online survey was in English therefore, most respondents who answered the questionnaire were literate. The employed individuals showed more acceptance towards vaccines in our survey, and the most likely reason is the prior availability of vaccines for the health care workers. The results are in agreement with another study where the healthcare workers received more vaccines than other people [37]. Furthermore, people being employed in any other profession depicted more acceptance towards taking vaccines than those with a low income [38]. The overall trend indicated that the respondents in phase 1a were highest as recommended by the CDC [39].

The available COVID-19 vaccines in Pakistan required two doses for complete effectiveness. The completion of the vaccine was critical for developing enough immunity to fight against the virus. Therefore, we inquired the respondents about the completion of the vaccine. It was found that more than 50% population had both doses of the vaccines administered, while 46.8% of respondents had one dose administered only. The data indicates that a large number of vaccine recipients were responsible enough to complete the course. The results were in line with another survey conducted in the U.S. which monitored the vaccine administration over the period of three months, and more than 60% population completed received both doses and completed the vaccine course [40]. The literature suggests that one dose is not enough for those who have not been previously infected with the disease; therefore, they need another shot as an immune booster [41]. According to this data, the number of single-dose vaccine recipients is sufficiently high. The participants who have been previously infected with the SARS-CoV-2 were almost 30%, and their vaccination should be completed on time. Although infection to SARS-CoV-2 generates an immune response and a single dose acts as a booster for them, but the antibodies are neutralized after a while, so another injection is needed to protect them against a lethal SARS-CoV-2 infection [42]. The number of males and females who received vaccines was comparable, and no gender discrimination was observed in our study. However, a few studies presented different intentions between males or females to receive the vaccine [42, 43].

The impact of pre-existing illnesses could alter the effectiveness of the COVID-19 vaccines. The evidence suggests that patients with comorbidities such as diabetes, hypertension, obesity, respiratory disorders such as asthma, COPD, or severe respiratory allergy are at a greater risk of mortality [44]. It is important to understand the biological mechanisms involved in SARS-CoV-2 infection and the immune system’s strength to lay out the preventive strategies against it. Upon inquiry from our participants, 31.7% responded that they were suffering from specific comorbidities that could aggravate the COVID-19. Among them, hypertension, diabetes, obesity, and asthma were the most common diseases. Hypertension has been reported in multiple studies as the foremost pre-existing comorbidity [45–47]. In another study focused on the health impact of comorbidities associated with COVID-19 in developing countries, similar results were presented, and hypertension was the commonly reported non-communicable disease followed by diabetes and obesity [48]. Although its underlying mechanism of contributing towards the severity of SARS-CoV-2 infection is unclear, it is frequently accepted that the patients suffering from hypertension and other cardiovascular diseases and are often suffering from a poor health status. According to our data, a large number of respondents who received the vaccines belonged to the older age group, and it is fairly common to be suffering from such ailments in the older population [49].

Obesity and diabetes have also been reported in many studies as pre-existing comorbidity in the patients of COVID-19 [50–52] which is in line with our results. There are numerous reasons due to which diabetic and obese patients are likely to develop COVID-19 infection. The susceptibility to hyperinflammation increases in diabetic patients and the development of cytokine storms. Studies reported a significant increase in the CPR and IL-6 levels in COVID-19 patients who had diabetes [53]. Similarly, administration of DPP4 inhibitors can weaken the innate immune response in COVID-19 patients because they exhibit their anti-inflammatory effect and reduce macrophage infiltration [54]. According to our results, mostly obese participants were females because obesity is more common among females in Pakistan [54]. Similarly, only a small percentage of respondents were male smokers, because according to the socio-demographic pattern of smoking, males are significantly frequent cigarette smokers than females [55, 56]. Smokers were found reluctant in getting vaccination. United States of America conducted a survey specifically to evaluate response of smokers regarding vaccine and found 0nly 49.1% were willing to get the dose where and 24.9 were not sure and 26% rejected to get vaccinated [57].

Our survey continues to present the results that injection of the vaccine also caused certain signs and symptoms to appear. More than 50% of participants responded that they experienced symptoms such as pain and swelling at the injection site, redness, and rash. Similar results were obtained by a survey performed in India, where the respondents presented similar complaints [58]. In other symptoms experienced in the whole body, the most commonly reported symptoms were experiences mostly after receiving a second dose. These include general weakness, muscle pain, dizziness, nausea/vomiting, headache, and fever. In contrast to our results, fever was the most commonly reported symptom in a study, followed by breathlessness and flu-like symptoms [59]. Another survey reporting the side effects of the Sinopharm vaccine, which was allocated by the Pakistani government to be administered, presented very similar results to ours, where weakness, fatigue, and pain at the injection site were among the commonly reported symptoms in the vaccine recipients of age ≤49 years and >49 years old, especially after receiving the second dose [60]. The occurrence of symptoms was observed to be increasing with age, which is plausible as the underlying poor health conditions can aggravate the symptoms, as discussed earlier.

The COVID-19 vaccine acceptance hesitancy was reportedly common throughout the world [26]. This trend continues in Pakistan as well which has a foundational basis of many factors such as socio-demographic factors, religious and cultural beliefs along with low literacy rate and lack of understanding and knowledge of how the vaccines work [32]. According to the results obtained by our survey, less than 30% of people responded that their family members had not been vaccinated. The possible reasons could be the unavailability of the vaccination for the entire country’s population at the time of the survey. However, the above-mentioned possibilities cannot be ruled out. On the other hand, the overall acceptance in phase 1a was better than others. Similar results were reported by another study conducted in Pakistan, where 70% of healthcare workers showed acceptance towards getting vaccinated against COVID-19 [17]. The travel history of participants within the country showed that 30% of participants traveled after getting vaccinated, while more people had traveled before the vaccination. The fear of the COVID-19 3^rd^ wave outbreak was imminent, and the country was in a partial lockdown, and that could be the possible reason for less traveling [61].

Getting the vaccination against COVID-19 is no longer going to be an option or a matter of choice. However, the results of our studies presented a positive attitude towards getting vaccinated by choice, where more than 70% of participants responded that they had complete information on the vaccine, and most of them got vaccinated by choice. The reason could be a considerable number of healthcare workers and the literate population were included in the survey, so they out to have a better understanding of the health and social implications of getting vaccinated, which has been reported by other studies as well [1, 62]. Although almost 40% of participants of our study believe that the COVID-19 virus will stay here, almost double the participants did not discourage others to get vaccinated. Similar results were obtained in an online poll in the USA, where most people are still making up their minds about taking the vaccines, they appeared to have a likelihood of taking it soon which is encouraging and motivating for the general public [1].

## Conclusion

The COVID-19 virus has long become a global pandemic that continues to bring about chaos in lives and the global economy. A COVID-19 vaccine represents a flame of hope for its prevention and ultimately, complete eradication. The present study investigated the attitude, knowledge, and perception of Pakistani people towards COVID-19 vaccines. The findings conclude that participants had inadequate knowledge but a positive perception and attitude towards getting vaccinated. It is suggested for the country’s policymakers to ensure the dissemination of adequate scientific and social knowledge towards the general public and thorough availability of the vaccines so that they can benefit fully from the opportunity.

### Limitations

Most of the survey was online, and the questionnaire was in English; hence, it was not possible to include illiterate people. The online self-reporting method may be subjected to memory biases or untrue information provided by the respondents. Despite participation of 503 people in the study, this study can’t be considered as general perception of all socio-economic groups that reside in different provinces of the Pakistan as majority of participants were from Punjab. This survey was conducted soon after the initiation of mass vaccination of the eligible population of Pakistan, and its findings may change after the complete implementation of the vaccine program.

### Future perspective

This study is among the first about community perception about COVID-19 vaccinations in Pakistan. Its findings will be crucial for the health policymakers to remove the barriers to rapid vaccination of the highest possible percentage of the Pakistani population to alleviate the negative impacts of the pandemic on the country.

## Supporting information

S1 Data (CSV)
